# Facile Synthesis of cis-Dichloro-(1,4,7-Triazacyclononane) Platinum(II) and its Screening Against Human Ovarian Cancer (SK-OV-3) and Leukemia (K-562) Cell Lines

**DOI:** 10.1155/MBD.2002.61

**Published:** 2002

**Authors:** Robert I. Haines, Aleisha M. Murnaghan

**Affiliations:** 1 Department of Chemistry University of Prince Edward Island 550 University Avenue Charlottetown PE CIA 4P3 Canada

## Abstract

A simple method for the preparation of *cis*-dichloro(1,4,7-triazacyclononane)platinum(II), *cis*-Pt(tacn)Cl_2_ is presented, together with the results of screening the compound against the K-562 (leukemia) and SK-OV-3 (ovarian) human cancer cell lines. While the compound shows no activity against K-562 cells, there is evidence for some cytotoxicity against SK-OV-3. The compound is much less effective than cisplatin, and its limited solubility restricts the useable concentration range.


### Facile Synthesis of cis-Dichloro-(1,4,7-Triazacyclononane) Platinum(Ii) and its Screening Against Human Ovarian Cancer (SK-OV-3) and Leukemia (K-562) Cell Lines.

Robert I. Haines* and Aleisha M. Murnaghan

*Department of Chemistry, University of Prince Edward Island, 550 University Avenue, Charlottetown, PE, C1A 4P3, Canada.*

### ABSTRACT

A simple method for the preparation of *cis*-dichloro(1,4,7-triazacyclononane)platinum(II), *cis*-$Pt(tacn)Cl_2$ is presented, together with the results of screening the compound against the K-562 (leukemia) and SK-OV-3 (ovarian) human cancer cell lines. While the compound shows no activity against K-562 cells, there is evidence for some cytotoxicity against SK-OV-3. The compound is much less effective than cisplatin, and its limited solubility restricts the useable concentration range.

### INTRODUCTION

While the matriarchal platinum anti-cancer agent, cisplatin, has been known for 150 years, its efficacy in the treatment of a variety of types of cancer was only discovered serendipitously by Rosenberg in the 1960's /1-3/. Since its approval for clinical use in 1978, it has developed into a $500 M a year industry, and has proved very successful in the treatment of testicular and ovarian cancer, as well as contributing to the treatment of lymphomas, head and neck, and bladder cancer /4/. However, the clinical use of cisplatin has several drawbacks. These include severe nausea and vomiting and severe nephrotoxicity /5/. While these problems have been ameliorated by aggressive diuresis and the use of anti-emetics, a "second-generation" of platinum anti-cancer agents were sought /6/. In these platins, chloro- ligands were replaced by more stable chelating ligands, resulting in complexes that were found to have lower renal toxicity, while retaining anti-tumour activity /7/. A key example is carboplatin, which causes significantly less nausea, vomiting and nephrotoxicity than cisplatin, but is as effective in the treatment of ovarian cancer /8/.

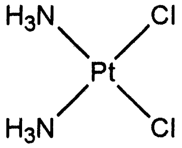

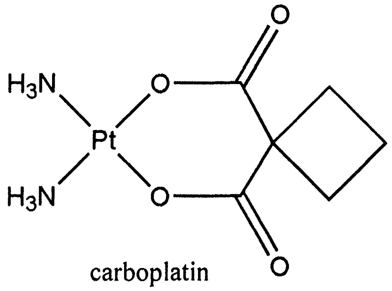

cisplatin carboplatin

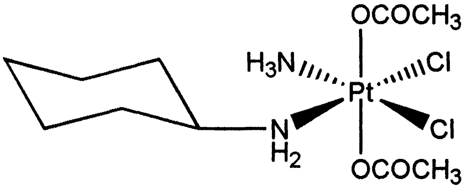

JM 216

These platins still require intravenous administration, and in the 1990's a "third generation" of platins were explored. These are based on platinum(IV) compounds, that, having a $d^6$ low-spin electronic configuration, are substitutionally inert. Hence these complexes may be resistant to hydrolysis by gastric juices, and may be orally viable. One example is JM 216. It is believed that once JM216 enters a cell, native anti-oxidants, such as cysteine or methionine present in cell proteins reduce the octahedral Pt(IV) complex to the square-planar Pt(II) form. Thus acetato- ligands are lost and a cisplatin-like molecule is generated *in situ*. The evolution of platin chemistry has been thoroughly reviewed periodically /9-11/ and the chemistry and biochemistry of cisplatin and its successors have been elegantly detailed in a recent book /12/.

Our laboratory has been interested in the structure and redox activity of transition metal macrocycles /13/. We have recently, turned our attention to the investigation of platinum complexes of *meso-* and *rac-*macrocycles bearing pendant carboxylato- arms, to determine whether these macrocycles stabilize platinum(IV) complexes, that will be substitutionally inert, and that may be reduced by cysteine to the platinum(II) form /14/. In that study we found that the great thermodynamic stability of the platinum(IV) complexes, imparted by the "macrocyclic effect" /15/ and by the negatively-charged pendant arms induced disproportionation of the platinum(II) compounds (formed by reduction of the Pt(IV) forms by cysteine), resulting in the precipitation of platinum(0) metal, free macrocycle and reformation of the platiunum(IV) complex. Thus these compounds were deemed not to be viable candidates as anti-tumour drugs. Consequently, we are presently examining the anti-cancer potential of platinum macrocycles where the propensity for disproportionation is avoided.Hence the present work examines the anti-cancer potential of the title compound, *cis*-dichloro-(1,4,7-triazacyclononane)platinum(II), *cis*-Pt(tacn)$Cl_2$.

*cis*-Pt(tacn)$Cl_2$.

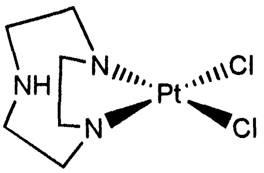

The tacn macrocycle is too small to encapsulate a central metal ion, thus no disproportionation is expected, since the platinum ion is coordinated in an exocyclic fashion. We report here a facile synthesis of the title compound, together with the results of its screening against two human cancer cell lines, K-562 (leukemia) and SK-OV-3 (ovarian).

### MATERIALS AND METHODS

Potassium tetrachloroplatinate(II), 3-(4,5-dimethylthiazol-2-yl)-2,5-diphenyltetrazolium bromide, RPMI 1640 medium (without L-glutamine), McCoy's 5a (without L-glutamine), and cisplatin were obtained from Sigma. L-Glutamine and fetal bovine serum were obtained from CanSera. Molecular Biology grade DMSO was obtained from Fisher. 1,4,7-Triazacyclononane (tacn) was synthesized by a modified literature method /16/. Its purity was confirmed by $^{1}H$ and $^{13}C$ NMR spectrometry. The K-562 cells were generously donated by Dr. Alastair Cribb. The SK-OV-3 cells were purchased from American Type Culture Collection.

Fourier transform infrared spectra were measured using a Bruker Equinox 55 spectrometer. Solution NMR spectra were measured using a Bruker Avance 300MHz spectrometer. Solid state spectra were run at the Atlantic Regional NMR facility, Dalhousie University. Platinum analyses were carried out using a Varian SpectrAA-10 atomic absorption spectrometer.

### Synthesis

To a stirred, aqueous solution (30 mL) of $K_2PtCl_4$ (3.1g, 7.5 mmol) was added dropwise, under nitrogen, a methanolic solution (10 mL) of tacn (0.95g, 7.4 mmol). The $K_2PtCl_4$ solution was filtered prior to reaction, to remove any metallic platinum and $K_2PtCl_6$ impurities. The solution was stirred at room temperature for one hour, during which time a bright orange precipitate formed. The mixture was allowed to stir, in the dark, overnight and then filtered. The precipitate was washed with cold water, methanol and dried *in vacuo*. Yield 1.36 g (47 %). Anal. Found: Pt 50.8 % Calcd. for $PtC_6H_{15}N_3Cl_2$:Pt 49.4 %. IR (KBr $cm^{-1}$): 3489 (NH), 3159 ($CH_2$), 1620 (NH), 1448 ($CH_2$), 1352 (CN), 1129 (CN), 1054 (CN). The $^{13}C$ MAS NMR (solid state) gave a broad peak at 54.77 ppm.

### Solubility of *cis*-Pt(tacn)$Cl_2$

An excess of solid *cis*-Pt(tacn)$Cl_2$ was shaken with 9% w/v saline solution and thermostated at 25°C in a recirculating water bath. The saturated solution was filtered and analysed for platinum concentration by Atomic Absorption spectrometry. The saturated solution was found to contain 704 ppm of platinum, equating to a molar solubility of $3.6 \times 10^{-3}$ mol $dm^{-3}$.

### *In vitro* screening

The cell lines tested were K-562 (leukemia) and SK-OV-3 (ovarian). The K-562 cells were grown in RPMI 1640 culture medium and the SK-OV-3 cells were grown in McCoy's 5a culture medium. The media were supplemented with 10% fetal bovine solution and 2% L-glutamine prior to use. The flask containing the cells was incubated at 37°C under an atmosphere of 5.0 % $CO_2$ overnight. The cells were seeded at concentrations of 40,000, 20,000 and 10,000.cells per well (for K-562) and 80,000, 40,000 and 20,000 cells per well (SK-OV-3) for the 24, 48 and 72 hour assays respectively in 96-well microtitre plates. These cell densities fell within the linear response curve for the MTT viability assay.

The platinum compound was dissolved in 9% w/v saline solution prior to use, and filtered through 0.22

μm cellulose acetate filters to produce a sterile solution. The solution was serially diluted with the culture medium and 20 μL aliquots were added to the wells containing 180 μLof cells so that the final concentrations in the wells ranged from 0 to 300 μM. This range was limited by the low solubility of the compound.

### MTT Assay

Cell viability was measured as the activity of the mitochondrial succinate dehydrogenase /17/. To each cell was added 20 μL of 5 mg $mL^{-1}$ MTT (3-(4,5-dimethylthiazol-2-yl)-2,5-diphenyltetrazolium bromide) solution and the plates incubated at 37°C for 2 hours. After addition of 100 μL of biology grade DMSO, with agitation, the absorbance of the resulting purple formazan solution was measured, using a Molecular Devices SpecMax 343 diode array spectrometer, running the Softmax Pro software package. The percentage of viable cells was calculated by comparing the absorbance of the platinum-treated cells with that of the untreated controls.

### RESULTS AND DISCUSSION

Early studies on the complexation between platinum(II) and tacn /18/ showed the interaction to be strongly pH dependent. At low pH, the macrocycle was protonated, and the tetrachloroplatinate(II) salt, $[tacnH_2](PtCl_4)$ precipitated from solution as a pink solid. By raising the pH and adding bromide ion, the *cis*-dibromo complex, *cis*-$Pt(tacn)Br_2$ was formed as a yellow product. In a recent study /19/ Hambley *et al.* isolated the protonated analogue, $[cis\text{-}Pt(tacnH)Cl_2]^+$ as the tetrachloroplatinate(II) salt, and reported the crystal structure. In the present work, we reacted the free macrocyclic triamine with potassium tetrachloroplatinate in mixed-aqueous medium. Under these conditions, where the solution pH is high, no protonated macrocycle was produced, and the requisite *cis*-dichloro product, *cis*-$Pt(tacn)Cl_2$ was produced directly. The low solubility of the complex afforded ready precipitation, while the potassium chloride side-product remained in solution. The $^1H$ NMR showed a single peak at 2.23 ppm, suggesting that the compound is fluxional in solution. Similar fluxional behaviour has been observed for palladium complexes of tacn /20/. The low solubility of the compound prevented the measurement of a $^{13}C$ NMR spectrum in solution. However, a solid state $^{13}C$ spectrum was run (at Dalhousie U.). A single broad peak, centred at 54.77 ppm was obtained. The broadness of the peak may be explained by the nature of the solid sample being amorphous.

### *In vitro* screening

Table 1 shows the percent survival for K-562 cells after exposure to *cis*-$Pt(tacn)Cl_2$ for (a) 24, (b) 48 and (c) 72 hours, for concentrations of platinum compound ranging from 3 x $10^{-6}$ to 3 x $10^{-3}$ mol $dm^{-3}$. The values presented are the average of at least four sets of replicate measurements. The raw data for all replicates, including absorbance data for the formazan solutions and the calculated % survivals have been provided as supplementary information. It is seen that *cis*-$Pt(tacn)Cl_2$ is ineffective in causing apoptosis in K-562 cells.

**Table 1**

Average % cell survival for cytotoxicity assays for K-562 cells treated with *cis*-Pt(tacn)$Cl_2$, for a series of concentrations of the compound, for 24, 48 and 72 hours.

| [*cis*-Pt(tacn)$Cl_2$] (mol $dm^{-3}$) | 24 hour exposure | 48 hour exposure % survival | 72 hour exposure |
|---|---|---|---|
| blank | 100 (100) | 100 (100) | 100 (100) |
| $1.0 \times 10^{-8}$ | 95 (91) | 93 (95) | 95 (104) |
| $1.0 \times 10^{-7}$ | 102 (91) | 96 (99) | 103 (114) |
| $1.0 \times 10^{-6}$ | 107 (97) | 97 (102) | 106 (100) |
| $1.0 \times 10^{-5}$ | 100 (87) | 87 (66) | 104 (53) |

[a] values in parentheses refer to cisplatin control results

This is not unexpected, since platinum-based anti cancer drugs have traditionally been found to be effective against solid tumour cancers /21/. The K-562 leukemia cells are of the soft-tissue type. The control experiments, using cisplatin, showed a similar lack of apoptosis in K-562 cells, although there is some indication of effectiveness after 72 hours exposure.

Table 2 shows the percent survival for SK-OV-3 cells after exposure to *cis*-Pt(tacn)$Cl_2$ for (a) 24, (b) 48 and (c) 72 hours, for concentrations of platinum compound ranging from $3 \times 10^{-6}$ to $3 \times 10^{-3}$ mol $dm^{-3}$. Little cell-death was observed after 24 and 48 hours exposure. However, after 72 hours, the average percent survival for cells exposed to $3 \times 10^{-6}$, $3 \times 10^{-5}$ and $3 \times 10^{-4}$ mol $dm^{-3}$ platinum compound was 85, 67 and 77% respectively. This may be compared to cisplatin, where corresponding survival percentages were 75, 14 and zero respectively. Thus we may conclude that while *cis*-Pt(tacn)$Cl_2$ shows some efficacy for apoptosis in ovarian cells, it is much less effective than cisplatin. This is somewhat surprising, since the complex contains the well-recognised important "*cis*-dichloroplatinum(II)" moiety, analogous to cisplatin. Further, its limited

**Table 2**

Average % cell survival for cytotoxicity assays for SK-OV-3 cells treated with *cis*-Pt(tacn)$Cl_2$, for a series of concentrations of the compound, for 24, 48 and 72 hours.

| [*cis*-Pt(tacn)$Cl_2$] (mol $dm^{-3}$) | 24 hour exposure | 48 hour exposure % survival | 72 hour exposure |
|---|---|---|---|
| blank | 100 (100) | 100 (100) | 100 (100) |
| $6.0 \times 10^{-7}$ | 103 (85) | 105 (89) | 85 (75) |
| $6.0 \times 10^{-6}$ | 102 (80) | 89 (51) | 67 (14) |
| $6.0 \times 10^{-5}$ | 110 (73) | 98 (2) | 77 (0) |
| $6.0 \times 10^{-4}$ | 119 (67) | 98 (0) | 82 (0) |

[a] values in parentheses refer to cisplatin control results solubility in saline solution further inhibits its use. Current work in our laboratory is directed towards increasing the lypophilicity of platinum-tacn complexes by appending aliphatic tails to the macrocycle. This should serve to increase the bioavailability of the compound. Use of cyclodextrins as drug-carriers will address the solubility problems. This work is ongoing, and will be reported later.

### ACKNOWLEDGEMENTS

We are grateful to the Natural Science and Engineering Research Council (NSERC) of Canada and the PEI Cancer Research Council for financial support. Thanks too go to the University of Prince Edward Island Senate Committee on Research for a research grant. We are indebted to the Canadian Foundation for Innovation (CFI), the Atlantic Canada Opportunities Agency (ACOA) and the Levesque Foundation for the purchase of infrastructure equipment.

### REFERENCES

1. B. Rosenberg, L.V. Camp, T. Krigas, *Nature*, **205**, 698-699 (1965).
2. B. Rosenberg, L.V. Camp, J.E. Trosko, V.H. Mansour, *Nature*, **222**, 385-386 (1969).
3. B. Rosenberg, L.V. Camp, *Cancer Research*, **30**, 1799-1802 (1970).
4. M.C. Christian, *Semin. Oncol.*, **19**, 720-733 (1992).
5. P.J. Loehrer, L.H. Einhorn, *Ann. Intern. Med.*, **100**, 704-713 (1984).
6. T.A. Connors, M. Jones, W.C. Ross, P.D. Braddock, A.R.Khokhar, M.L. Tobe, *Chem. Biol. Inter.*, **5**, 415-424 (1972).
7. K.R. Harrap, *Cancer Treat. Rev.*, **12**, 21-33 (1985).
8. D.S. Alberts, S. Green, E.V. Hannigan, R. O'Toole, D. Stock-Novack, P. Anderson, A. Surwite, V.K. Malvlya, W.A. Nahas, C.J. Jolles, *J. Clin. Oncol.*, **10**, 706-717 (1992).
9. M.J. Cleare, *Coord. Chem. Rev.*, **12**, 349-405 (1974).
10. S.E. Sherman, S.J. Lippard, *Chem. Rev.*, **87**, 1153-1181 (1987).
11. T.W. Hambley, *Coord. Chem. Rev.*, **166**, 181-223 (1997).
12. E. Wong, C.M. Giandomenico, *Chem. Rev.*, **99**, 2451-2466 (1999).
13. J. Burgess, J. Fawcett, R.I. Haines, K. Singh, D.R. Russell, *Transition Metal Chem.*, **24**, 355-361 (1999); R.I. Haines, D.R. Hutchings, R.J. Lucas and D. Miller, *Can. J. Chem.*, **79**, 54-62 (2001); R.I. Haines and R. Baldwin, *Transition Met. Chem.*, **27**, 284-289 (2002).
14. R.I. Haines, D.R. Hutchings and T.M. McCormack, *J. Inorg. Biochem.*, **85** (2001) 1-7.
15. L.F. Lindoy, "*The Chemistry of Macrocyclic Ligand Complexes*", Cambridge University Press, 1989, Chap. 2.
16. G.H. Searle and R.J. Geue, *Aust. J. Chem.*, **37**, 959-970 (1984).
17. T. Mosmann, *J. Immunological Methods*, **65**, 55-64 (1983).
18. K. Weighardt, M. Koppen, W. Swiridoff and J. Weiss, *J. Chem. Soc., Dalton Trans.*, 1869-1872 (1983).

19. M.S. Davies, R.R. Fenton, F. Huq, E.C.H. Ling and T.W. Hambley, *Aust. J. Chem.*, **53**, 451-456 (2000).
20. B. Chak, A. McAuley, T.W. Whitcombe, *Can. J. Chem.*, **72**, 1525-1532 (1994).
21. B. Lippert (ed.),"*Cisplatin: Chemistry and Biochemistry of a Leading Anticancer Drug*", Verl. Helv. Chim. Acta; Weinheim: Wiley-VCH, 1999.

